# Treatment of Jumper’s Knee with Extracorporeal Shockwave Therapy: A Systematic Review and Meta-Analysis

**DOI:** 10.2478/hukin-2022-0089

**Published:** 2022-11-08

**Authors:** Magdalena Stania, Tomasz Król, Wojciech Marszałek, Justyna Michalska, Piotr Król

**Affiliations:** 1 Institute of Sport Sciences, Academy of Physical Education, Katowice, Poland.; 2 Department of Kinesitherapy and Special Methods, School of Health Sciences in Katowice, Medical University of Silesia, Katowice, Poland.

**Keywords:** patellar ligament, physical therapy modalities, musculoskeletal system, athletes

## Abstract

The aim of this systematic review and meta-analysis was to determine the therapeutic efficacy of extracorporeal shockwave therapy (ESWT) for athletes with patellar tendinopathy. We searched PubMed, EBSCOHost and Ovid for randomized controlled trials (RCTs) which evaluated the therapeutic efficacy of ESWT in athletes with jumper’s knee. The methodological quality of RCTs was rated with the Physiotherapy Evidence Database scale. Data in the meta-analysis were expressed as standardized mean difference (SMDs) and 95% confidence intervals (CIs). Heterogeneity was assessed with I^2^ statistics. Of 192 records identified, a total of seven articles met the inclusion criteria. The ESWT and control groups with any other conservative treatment did not differ significantly with respect to the Visual Analogue Scale (VAS) long-term scores obtained at ≥ 6 months of therapy completion (SMD: -0.33; 95% CI: -4.64 to 3.98; p = 0.87; I^2^= 98%). Furthermore, no significant differences were found between the ESWT and control groups regarding the pooled Victorian Institute of Sports Assessment for Patella (VISA-P) scores for long-term outcomes (SMD: 8.21; 95% CI: -39.3 to 55.73; p = 0.73; I^2^= 99%). The ESWT and control groups did not differ significantly on the VAS and VISA-P scores for long-term outcomes. In both cases, heterogeneity was considered to be high. Hence, no clear and generalized conclusions can be drawn regarding ESWT effectiveness in athletes with patellar tendinopathy.

## Introduction

Patellar tendinopathy (commonly called jumper’s knee) is a degenerative condition of the knee extensor mechanism which is caused by overloading and accumulation of microinjuries (Rudavsky and Cook, 2014). A literature review has found that horizontal landing poses the greatest risk for patellar tendinopathy (Van der Worp et al., 2014). Hence, athletes that jump frequently during competition and practice are at an increased risk of developing patellar tendinopathy with the overall prevalence from 8.5 to 14.2% (Lian et al., 2005; Zwerver et al., 2011). The type of sports training also affects the prevalence of jumper’s knee (Lian et al., 2005). The highest prevalence was found among volleyball players, i.e., 14.4% and 44.6% in Lian et al.’s (2005) and Zwerver et al.’s (2011) studies, respectively, and basketball players (31.9%) (Lian et al., 2005). Non-jumping and repetitive low loading activities (e.g., swimming, running, cycling) do not put much stress on the patellar tendon and therefore, the risk of developing jumper’s knee is rather low (Rhind et al., 2022; Rudavsky and Cook, 2014).

Patellar tendinopathy reduces the quality of life and interferes with engagement in vigorous physical and frequently, also professional sports activity. Fifty-five per cent of active athletes with patellar tendinopathy report a negative impact on their sports performance, while 16% admit reduced work ability (de Vries et al., 2015). Clinical manifestations in athletes tend to be moderate, but long-lasting causing the sufferers to seek effective therapies (Kettunen et al., 2002) which would allow them to return to professional *sports* and physical *activity*.

Early symptoms of jumper’s knee are usually managed conservatively. Pain reduction involves physical interventions, e.g., laser therapy, sonotherapy or transfer energetic capacitive and resistive therapy (commonly referred to as TECAR therapy) (Costantino et al., 2005). Other therapeutic options include the use of infrapatellar straps (Dar and Mei-Dan, 2019) and platelet rich plasma, high-volume image-guided injections of saline (Abate et al., 2018). Conservative treatment unsuccessful after 3 to 6 month therapy may indicate a need for surgery (Rodriguez-Merchan, 2013).

Although extracorporeal shockwave therapy (ESWT) has long been used as a conservative treatment for patellar tendinopathy, its curative effect on degenerative changes within tendons has not been fully elucidated. Neither have clear-cut recommendations for professional and amateur athletes been developed regarding the optimal ESWT variables.

Therefore, the aim of the current systematic review and meta-analysis was to determine the therapeutic efficacy of ESWT in athletes with patellar tendinopathy. The research question was defined by the PICO-model in accordance with the updated guidelines for the Preferred Reporting Items for Systematic Reviews and Meta-Analyses (PRISMA) statement (Page et al., 2021): *Population*: athletes with patellar tendinopathy; *Intervention*: extracorporeal shockwave therapy; *Comparator*: sham treatment or any other conservative treatment; *Outcomes*: pain intensity assessed with the Visual Analogue Scale (VAS) for a *s*hort- and a long-term follow-up; patient-reported functional outcomes assessed with the Victorian Institute of Sport Assessment-Patella (VISA-P) for a *s*hort- and a long-term follow-up. We believe that conclusions of this review and meta-analysis will help athletes, coaches and physical therapists adhere to standard ESWT therapies for a variety of sports.

## Methods

### Data Sources and Searches

A search was conducted using the following databases: PubMed, EBSCOHost and Ovid according to the PRISMA statement (Page et al., 2021). The last search was carried out on the 5^th^ of June, 2021. Keywords including ‘patellar tendinopathy’, ‘tendinitis’, ‘tendinopathy’, ‘patellar tendon’, ‘enthesopathy’, ‘shockwave therapy’, ‘shock wave therapy’, ‘shock-wave therapy’, ‘extracorporeal shockwave’, ‘extracorporeal shockwave therapy’, ‘ESWT’, ‘focused extracorporeal shockwave’, ‘radial extracorporeal shockwave therapy’, ‘therapy’ and ‘treatment’ were used in various configurations. Reference lists of all the retrieved articles were manually checked for additional studies. The full search strategies for all databases, including any filters and limits used, are presented in Appendix A (Table A.1.).

**Table A.1:** Detailed search strategy.

**PubMed**	
(((patellar tendinopathy[tiab] or patellar tendinitis[tiab] or jumper’s knee[tiab] or entesopathy[tw]) AND (shock wave[tiab] or shock-wave[tiab] or shockwave[tiab] or shockwaves[tiab] or extracorporeal[tiab])) AND (therapy[tiab] or treatment[tiab]))’	
Filter applied: English	

**EBSCOhost**	
TX ( patellar tendinopathy or patella tendinopathy or jumpers knee or patellar tendinitis or patella tendinitis ) AND TX ( shockwave therapy or shock wave therapy or shock-wave therapy or extracorporeal shockwave ) AND TX ( therapy or treatment )	
Filters applied:	
- English	
- abstract available	

**Ovid MEDLINE(R) ALL**	
1	Patellar Ligament/ and Tendinopathy/ 356
2 (patellar tendinopathy or patellar tendinitis or jumpers knee).mp. [mp=title, abstract, original title, name of substance word, subject heading word, floating sub-heading word, keyword heading word, organism supplementary concept word, protocol supplementary concept word, rare disease supplementary concept word, unique identifier, synonyms] 707	
3 (shock wave or shock-wave or shockwave or shockwaves or extracorporeal).mp. [mp=title, abstract, original title, name of substance word, subject heading word, floating sub-heading word, keyword heading word, organism supplementary concept word, protocol supplementary concept word, rare disease supplementary concept word, unique identifier, synonyms] 55428	
4	2 and 3 62
5	limit 4 to english 57

### Study Selection

The papers were checked for relevant content and were included based on the following criteria: (1) full-text article published in English; (2) randomized controlled trials (RCTs); (3) trials in which athletes with patellar tendinopathy were treated with ESWT; (4) trials in which controls underwent sham treatment or any other conservative treatment (e.g., exercise, injections, physical therapy); (5) trials containing the comprehensive description of ESWT (the following application variables were reported: the number of shocks, frequency, energy flux density or pressure, the number of sessions, frequency of treatment).

**Figure 1:**
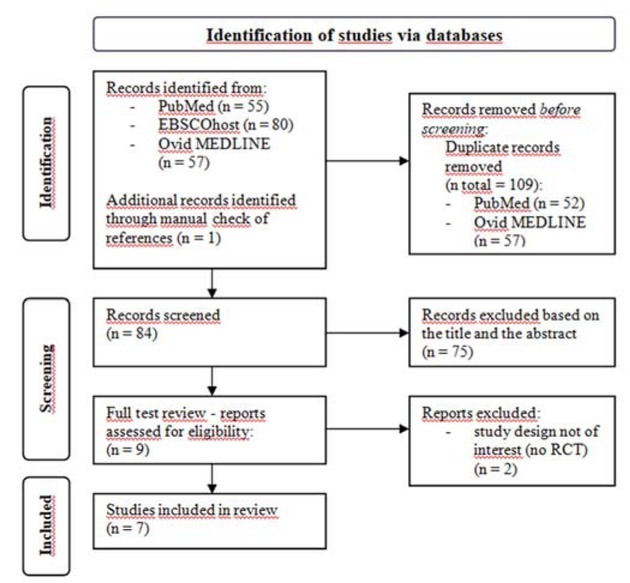
PRISMA 2020 diagram flowchart for the study search and selection.

We excluded trials using ESWT to treat knee cartilage disorders. Studies in animals were not included either. Conference abstracts, proceedings, case reports, commentary articles, secondary analyses, designs of RCTs, systematic reviews, meta-analyses and narrative reviews were also excluded (Stania et al., 2019). The results of the study selection procedure are summarized in the PRISMA 2020 flow diagram (Figure 1).

### Data Extraction and Quality Assessment

Three authors (MS, TK, WM) independently searched for articles, screened studies, and extracted data in a blinded manner. Any disagreements between the authors were resolved through consensus, with other research team members (JM and PK) acting as arbiters. Data were extracted from each study on participant characteristics, intervention (type of shock wave therapy, description of procedures), outcomes (primary and secondary outcomes, methods and timing of assessment) and results.

The methodological quality of randomized clinical studies was rated with the Physiotherapy Evidence Database (PEDro) scale. The PEDro scale (ranging from 1 to 10 points) is a valid measure of the methodological quality of randomized clinical trials (de Morton, 2009).

Based on the PEDro score, the methodological quality of trials is rated as high (PEDro scores ≥7), medium (4 to 6), or low (PEDro scores ≤ 3) (Liao et al., 2018). The methodological quality of the included articles was assessed by three reviewers (MS, TK, WM). In the event of disagreement, consensus was sought by involving another researcher (JM).

### Meta-analysis of effect estimates

Meta-analysis was undertaken where data were available from more than one study assessing the same outcome. The analysis included original papers which compared the outcomes of ESWT (as a monotherapy) with those of other conservative (non-surgical) therapies. Two outcomes were taken into consideration, i.e., pain reduction assessed with the Visual Analogue Scale and functional improvement verified using the Victorian Institute of Sports Assessment for Patella (VISA - P). Analyses were carried out for long-term scores obtained at ≥ 6 months of therapy completion (a long-term follow-up). It was not possibile to undertake the meta-analysis for the short-term scores as there was only one study which met the inclusion criteria.

Data in the meta-analysis were expressed as standardized mean differences (SMDs) and 95% confidence intervals (CIs). Heterogeneity was assessed with *I^2^* statistics: 25% was considered low, 50% moderate, and 75% high. Statistical significance was set at *p* < 0.05. The random effects model was applied for the pooled effect estimates.

## Results

### Search Results

The search and databases yielded a total of 192 articles, of which only 7 met the inclusion criteria (Figure 1). The total pooled sample size of all of the included studies was 155 participants in the experimental group and 156 participants in the control group.

### Quality Assessment

Table 1 summarizes the methodological quality of randomized clinical trials included in our review that were rated with the PEDro scale. The methodological quality of randomized clinical trials included in this review was rated as high (Lee et al., 2020; Vetrano et al., 2013; Zwerver et al., 2011) or medium (Cheng et al., 2019; Taunton et al., 2003; Wang et al., 2007).

**Table 1:** Randomized clinical trials and the effectiveness of ESWT for patellar tendinopathy rated with the Physiotherapy Evidence Database (PEDro) scale.

Reference	1	2	3	4	5	6	7	8	9	10	11	Score*
**Taunton et al., 2003**	+	+	-	+	+	-	-	+	-	+	-	**5**
**Wang et al., 2007**	**+**	**+**	**-**	**+**	**-**	**-**	**+**	**+**	**-**	**+**	**+**	**6**
**Zwerver et al., 2011**	+	+	+	+	+	-	+	+	+	+	+	**9**
**Vetrano et al., 2013**	+	+	-	+	-	-	+	+	+	+	+	**7**
**Thijs et al., 2017**	+	+	+	+	+	-	+	+	+	+	+	**9**
**Cheng et al., 2019**	+	+	-	+	-	-	-	-	-	+	+	**4**
**Lee et al., 2020**	+	+	+	+	+	-	-	+	+	+	+	**8**

1: Eligibility criteria specified; 2: Subjects randomly allocated to groups; 3: Allocation concealed; 4: Groups similar at baseline; 5: Blinding of all subjects; 6: Blinding of all therapists; 7: Blinding assessors; 8: >85% follow up; 9: Intention-to-treat analysis; 10: Between-group statistical comparison; 11: Point and variability measures. *Eligibility criteria item is not included in PEDro score calculations

### Results of meta-analysis

**Figure 2:**
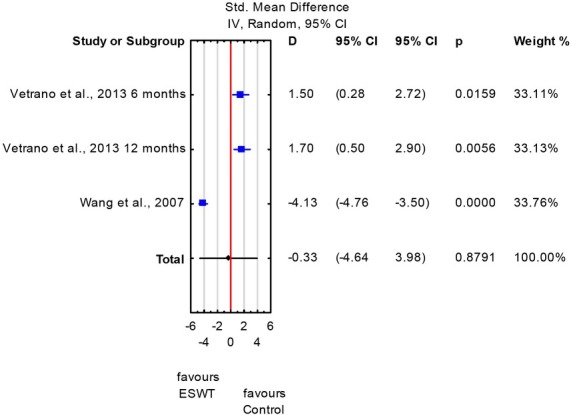
Forest plot of visual analogue scale (VAS) scores – measurements at ≥ 6 months of therapy completion - ESWT and control groups.

The meta-analysis did not reveal significant differences between the ESWT and control groups regarding the Visual Analogue Scale long-term scores obtained at ≥ 6 months of therapy completion (SMD: -0.33; 95% CI: -4.64 to 3.98; *p* = 0.87) (Figure 2). However, significant heterogeneity was observed with respect to longterm pain reduction (*I^2^*= 98%, *p* < 0.0001).

**Figure 3:**
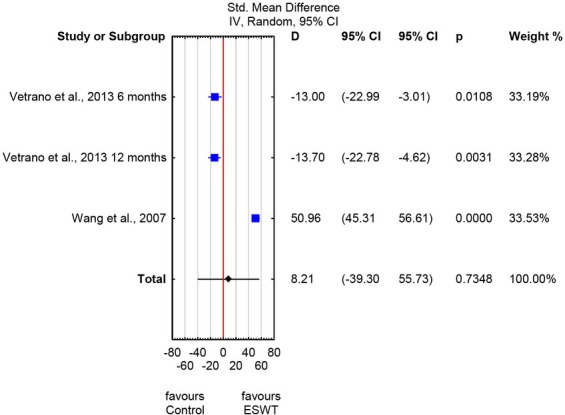
Forest plot of Victorian Institute of Sports Assessment–Patella (VISA-P) scores – measurements at ≥ 6 months of therapy completion - ESWT and control groups.

No significant differences were also found between the ESWT and control groups on the pooled VISA-P scores in the long-term follow-up (SMD: 8.21; 95% CI: -39.3 to 55.73; *p* = 0.73; *I^2^*= 99%) (Figure 3).

## Discussion

According to our meta-analysis, pooled estimates for VAS and VISA-P scores of the longterm follow-ups did not differ significantly between the ESWT and control groups, with confidence intervals crossing the line of no effect (Figures 2 and 3). For most of the cases, heterogeneity was considered to be high (*I*^2^: 98-99%). Therefore caution should be taken when interpreting these results. Also, no clear and generalized conclusions can presently be drawn regarding ESWT effectiveness in athletes with patellar tendinopathy.

Studies with high quality PEDro scores revealed a reduction in subjective patellar tendinopathy complaints at one week of shockwave therapy in 65% of athletes (Zwerver et al., 2011). At the 6- and 12-month follow-up, the improvement was noted in 65.2% and 60.8% of athletes, respectively (Vetrano et al., 2013). However, although shockwave intervention proved to be an effective treatment of jumper’s knee, it did not turn out superior to placebo therapy (Zwerver et al., 2011) or platelet-rich plasma injections (Vetrano et al., 2013). A combination of eccentric exercises and extracorporeal shockwave therapy also failed to increase treatment efficacy in athletes with jumper’s knee (Lee et al., 2020; Thijs et al., 2017). Medium quality studies on jumper’s knee showed greater efficacy of extracorporeal shockwave therapy compared to the placebo intervention (Taunton et al., 2003) and other forms of conservative treatment (Wang et al., 2007). At the 2- to 3-year follow-up, the overall clinical results were excellent in 43%, good in 47% and fair in 10% of athletes treated with shockwave therapy (Wang et al., 2007).

Differences in therapeutic efficacy of ESWT for patellar tendinopathy might be attributed to a couple of determinants described below. Table 2 shows the main characteristics of each intervention and key conclusions of the authors studying the efficacy question.

**Table 2:** Studies on the effectiveness of extracorporeal shock wave therapy for patellar tendinopathy.

Ref.	Sample size	Groups	Intervention	Outcome measure	Follow-up
**Taunton et al., 2003**	20	I: ESWT II: placebo ESWT	FSWT: 2000 shocks; 0.17 mJ/mm^2^; 3 to 5 sessions	VISA; vertical jump test	3 to 4 weeks after the third treatment, 3 to 4 weeks after the fifth treatment, at the 12^th^ week after the last treatment session
**Wang et al., 2007**	50	I: ESWT II: traditional conservative treatment	FSWT: 1500 shocks; 0.18 mJ/mm^2^; 1 session	VAS; VISA; ultrasonographic examination	1, 3, 6 and 12 months
**Zwerver et al., 2011**	62	I: ESWT II: sham ESWT	FSWT: 2000 shocks; 4 Hz; 0.1–0.58 mJ/mm^2^; 3 sessions, once a week	VISA-P; VAS	1, 12, and 22 weeks
**Vetrano et al., 2013**	46	I: autologus platelet-rich plasma II: ESWT	FSWT: 2400 shocks; 0.17–0.25 mJ/mm^2^; 3 sessions at 48- to 72-h intervals	VISA-P; VAS; modification of the Blazina scale	2, 6 and 12 months
**Thijs et al., 2017**	52	I: ESWT + eccentric training II: placebo ESWT + eccentric training	FSWT: 1000 shocks; 4 Hz; 0.2 mJ/mm^2^; 3 sessions, once a week	VAS; VISA-P; Likert score	6, 12 and 24 weeks
**Cheng et al., 2019**	51	I: ESWT II: control group (acupuncture, ultrasonic wave, microwave therapy)	RSWT: 2000 shocks; 9-12 Hz; 1.5 – 3 bars; (corresponds to 0.09-0.27 mJ/mm^2^); 16 sessions, once a week	VAS; isokinetic muscle strength test	After therapy (16 weeks)
**Lee et al., 2020**	30	I: exercise group (eccentric exercise + sham ESWT) II: combined group (eccentric exercise + ESWT)	FSWT: 1500 shocks; 4 Hz; from 0.08 mJ/mm^2^to the level that the subject could maximally tolerate; 6 sessions, once a week	VAS; VISA-P; ultrasonography; dynamometry	After therapy (12 weeks)

ESWT – Extracorporeal Shock Wave Therapy; FSWT – focused shockwave therapy; RSWT – radial shock wave therapy; VAS – Visual Analog Scale; VISA-P – Victorian Institute of Sport Assessment–Patella questionnaire

### Athletes with patellar tendinopathy – characteristics

Differences in therapeutic efficacy of ESWT could be attributed to large diversity across research materials including elite athletes during the competitive season (Lee et al., 2020; Zwerver et al., 2011), athletes in their off-season (Vetrano et al., 2013), individuals engaged in recreational sports (Wang et al., 2007) as well as patients of hospitals or sports medicine centers (Thijs et al., 2017). Each of these populations may have different etiological factors, e.g., the mechanical load on the patellar tendon in elite and non-elite athletes is much greater than that of hospital patients. Therapeutic success of ESWT is thus more difficult to reach in athletic populations as indicated by the studies of Zwerver et al. (2011) and Vetrano et al. (2013). It is also important that athletes do not engage in intensive physical activity during the therapy period. Zwerver et al. (2011) suggested that the total load on the tendon resulting from the combination of ESWT-induced collagen disorganization and the mechanical overload from sports training might be too high and the time for recovery was insufficient. Consequently, some interference with potential regenerative effects of ESWT might be expected. Therefore, some researchers recommended that their athletes would not engage in physical activity during therapy and up to six weeks following therapy completion (Vetrano et al., 2013; Vulpiani et al., 2007; Wang et al., 2007).

Researchers mainly recruited athletes with a history of over 3-month chronic patellar tendinopathy (Lee et al., 2020; Taunton et al., 2003; Wang et al., 2007; Zwerver et al., 2011). Only Thijs et al. (2017) included those who had suffered for a period of eight weeks. Timely differentiation between acute and chronic tendinopathy is essential for diagnostic and therapeutic considerations (Gabel, 1999). Zwerver et al. (2011) did not show superiority of ESWT intervention over the placebo treatment for reduction of jumper’s knee symptoms. According to the authors ESWT might fail in athletes suffering from reactive tendinopathy or early tendon dysrepair in the continuum of tendon pathology. ESWT proved beneficial in the degenerative stage of tendon disease (Zwerver et al., 2011).

Williams et al. (2017) suggested that MRI should be performed in those athletes with refractory patella tendinopathy who failed conservative treatment. MRI scans help determine the location and involvement of the fat pad. If the scans reveal intratendon changes only, ESWT might be expected to yield good outcomes. Athletes with fat pad involvement should undergo arthroscopic debridement without ESWT.

Differences in the outcomes of ESWT might also be accounted for by differences in the initial severity of jumper’s knee symptoms as assessed with the Victorian Institute of Sport Assessment–Patella questionnaire (VISA – P) (Zwerver et al., 2011).

### Types of ESWT

Jumper’s knee has much more frequently been treated with focused extracorporeal shockwave therapy (Lee et al., 2020; Thijs et al., 2017; Zwerver et al., 2011) than radial ESWT (Cheng et al., 2019). Focusing of shockwaves on a small-volume tissue target requires an accurate lesion location by ultrasounds. Radial ESWT allows treatment of both well-localized and more extensive lesions (Król et al., 2009).

Van der Worp et al. (2014) compared the efficacy of radial (3 therapy sessions, 2000 pulses, 8 Hz, 2.4 bars) and focused ESWT (3 therapy sessions, 2000 pulses, 4 Hz, 0.12 mJ/mm^2^) in athletes with patellar tendinopathy. Both treatment approaches reduced pain complaints and improved athletes’ function. However, none of the methods proved superior.

While treating patellar tendinopathy, most researchers used three sessions of ESWT (Thijs et al., 2017; Vetrano et al., 2013; Zwerver et al., 2011), with a one-week interval in between (Cheng et al., 2019; Lee et al., 2020; Thijs et al., 2017; Zwerver et al., 2011). There were also shorter intervals of 2 days (Vetrano et al., 2013). Wang et al. (2007) only held one therapeutic session, while Cheng et al. (2019) sixteen sessions. The results of a meta-analysis revealed that duration of the intervention affected treatment results in athletes with knee tendinopathy and those with other soft tissue disorders as well (Liao et al., 2018). Long intervention periods (≥ 1 month) exhibited beneficial therapeutic effects of ESWT on reducing pain and improving functional outcomes, while shorter treatments were less effective irrespective of the type of the extracorporeal shockwave (Liao et al., 2018).

With regard to radial ESWT, the number of shocks per session was 2000 (Cheng et al., 2019; van der Worp et al., 2014); the pulse frequency was 8 Hz (van der Worp et al., 2014) or 9–12 Hz (Cheng et al., 2019). The number of focused ESWT pulses was 1500 to 2500 and the pulse frequency was 4 Hz (Lee et al., 2020; Wang et al., 2007; Zwerver et al., 2011). For neuromuscular dysfunction, the effect of shockwave therapy tended to be dose-dependent and caused symptom improvement over time (Wang et al., 2007). The intensity of radial shockwaves applied for patellar tendinopathy was 1.5–3 bars (Cheng et al., 2019; van der Worp et al., 2014), whereas in focused shockwave therapy, the energy flux density was 0.08–0.58 mJ/mm^2^(Lee et al., 2020; Taunton et al., 2003; Wang et al., 2007; Zwerver et al., 2011).

Vetrano et al. (2013) recommended that chronic tendinopathies should be treated with focused ESWT at an energy flux density of 0.08 to 0.17 mJ/mm^2^. A meta-analysis of randomized controlled trials also showed that athletes with knee tendinopathies and other soft tissue disorders treated with low-energy (below 0.2 mJ/mm^2^) focused shockwaves exhibited higher rates of treatment success and noticeable functional improvement compared to those who received high-energy treatments. The inverse results were observed for radial shockwave therapy (Liao et al., 2018).

Depending on jumper’s knee manifestations, focused extracorporeal shockwaves were typically applied to the most painful spot within the patellar tendon (Thijs et al., 2017; Wang et al., 2007; Zwerver et al., 2010, 2011), a maximum of two specific points of tenderness (Taunton et al., 2003) or on the bone-tissue junction of the patellar or tibial insertions of the patellar tendon (Peers et al., 2003; Vetrano et al., 2013). Radial shockwaves were directed onto the distal pole of the patella and patellar tendon. The area of maximal tenderness was treated circumferentially, starting at the most painful site (Furia et al., 2013).

Klonschinski et al. (2011) concluded that application of local anesthesia inhibited the effect of low-energy extracorporeal shockwave therapy on nociceptors and therefore, should not be used during extracorporeal shockwave therapies. Otherwise, low-energy extracorporeal shockwave therapy is usually quite well tolerated (Vetrano et al., 2013; Wang et al., 2007; Zwerver et al., 2011). Only rarely have adverse effects been observed during or after ESWT interventions. Some athletes may complain of transient post-therapy skin reddening, but no bruising (Vetrano et al., 2013), moderate pain that occurs during the treatment, but resolves following treatment completion (Peers et al., 2003) or transient numbness and hypoesthesia (Wang et al., 2007).

ESWT can be used as monotherapy (Taunton et al., 2003; Wang et al., 2007; Zwerver et al., 2011) or can be combined with other treatment modalities, e.g., eccentric exercises (Lee et al., 2020; Thijs et al., 2017; van der Worp et al., 2014). However, a combination therapy had no additional effect compared to the sham-shockwave/eccentric group (Thijs et al., 2017). Those authors also warned against using very intensive eccentric exercises in combination with ESWT (Thijs et al., 2017).

### Evaluations of ESWT efficacy

The efficacy of ESWT for patellar tendinopathy was mainly evaluated using subjective measures (Thijs et al., 2017; Vulpiani et al., 2007; Wang et al., 2007; Zwerver et al., 2011) such as the Visual Analog Scale for pain (VAS Pain) (Cheng et al., 2019; Furia et al., 2013; Lee et al., 2020; Peers et al., 2003) and the Victorian Institute of Sport Assessment–Patella (VISA – P) questionnaire (Lee et al., 2020; Wang et al., 2007; Zwerver et al., 2010, 2011). The VISA score is a reliable index of the severity of patellar tendinopathy and shows excellent short-term test-retest, and inter-tester reliability as well as good short-term stability (Visentini et al., 1998). It should be noted though that the Likert score (Thijs et al., 2017), the Blazine Scale and its modification (Vetrano et al., 2013; Vulpiani et al., 2007) and the Roles and Maudsley score (Furia et al., 2013; Peers et al., 2003) were also used.

Some other authors relied on objective measurements, including ultrasound examination, to evaluate the vascularization in the area of the patellar tendon (Taunton et al., 2003; Wang et al., 2007), dimension and thickness of the patellar tendon, the presence of edema or swelling within the tendon (Wang et al., 2007). Cheng et al. (2019) explored the effects of extracorporeal shockwave therapy in athletes with patellar tendinopathy using isokinetic strength tests. Tendon mechanical properties were also assessed with dynamometry (Lee et al., 2020).

Literature analysis clearly indicates a need for randomized clinical trials with objective measurements of ESWT outcomes in athletes with patellar tendinopathy.

## Limitations of the study

This study has some limitations. Firstly, our analysis included a limited number of studies investigating the therapeutic efficacy of ESWT in athletes with patellar tendinopathy. Secondly, excluding studies published in languages other than English could lead to language bias. Also, we might have missed randomized clinical trials published in different languages.

## Conclusions

Extracorporeal shockwave therapy is a safe and non-invasive treatment for patellar tendinopathy. Contrary to surgical intervention, this form of therapy is not related to major complications or side effects; also, the patients do not need to take time off work. However, our meta-analysis did not reveal significant differences between ESWT and control groups with respect to the VAS and VISA-P scores for long-term outcomes. Hence, no definite conclusions on extracorporeal shockwave therapy efficacy for jumper’s knee can be drawn. There is a need for high-methodological-quality randomized clinical trials that may facilitate clearcut recommendations regarding the methodology of ESWT interventions for patellar tendinopathy.
